# A phase I/II study of carfilzomib 2–10-min infusion in patients with advanced solid tumors

**DOI:** 10.1007/s00280-013-2267-x

**Published:** 2013-08-25

**Authors:** Kyriakos P. Papadopoulos, Howard A. Burris, Michael Gordon, Peter Lee, Edward A. Sausville, Peter J. Rosen, Amita Patnaik, Richard E. Cutler, Zhengping Wang, Susan Lee, Suzanne F. Jones, Jeffery R. Infante

**Affiliations:** 1 South Texas Accelerated Research Therapeutics (START), 4383 Medical Dr, Room 4042, San Antonio, TX 78229 USA; 2 Drug Development Program, Sarah Cannon Research Institute/Tennessee Oncology, PLLC, Nashville, TN USA; 3 Pinnacle Oncology Hematology, Scottsdale, AZ USA; 4 Clinical Research, Tower Cancer Research Foundation, Beverly Hills, CA USA; 5 Hematology/Oncology and Clinical Research, Marlene and Stewart Greenebaum Cancer Center, University of Maryland, Baltimore, MD USA; 6 Clinical Science, Onyx Pharmaceuticals, Inc, South San Francisco, CA USA; 7 Drug Metabolism and Pharmacokinetics, Onyx Pharmaceuticals, Inc, South San Francisco, CA USA; 8 Biology, Onyx Pharmaceuticals, Inc, South San Francisco, CA USA; 9 Present Address: Clinical Research, Providence Saint Joseph Medical Center, Burbank, CA USA

**Keywords:** Proteasome inhibitor, Carfilzomib, Solid tumors, Pharmacokinetics, Pharmacodynamics

## Abstract

**Purpose:**

Tolerability, pharmacokinetics (PK), pharmacodynamics, and antitumor activity of carfilzomib, a selective proteasome inhibitor, administered twice weekly by 2–10-min intravenous (IV) infusion on days 1, 2, 8, 9, 15, and 16 in 28-day cycles, were assessed in patients with advanced solid tumors in this phase I/II study.

**Methods:**

Adult patients with solid tumors progressing after ≥1 prior therapies were enrolled. The dose was 20 mg/m^2^ in week 1 of cycle 1 and 20, 27, or 36 mg/m^2^ thereafter. The maximum tolerated dose or protocol-defined maximum planned dose (MPD) identified during dose escalation was administered to an expansion cohort and to patients with small cell lung, non-small cell lung, ovarian, and renal cancer in phase II tumor-specific cohorts.

**Results:**

Fourteen patients received carfilzomib during dose escalation. The single dose-limiting toxicity at 20/36 mg/m^2^ was grade 3 fatigue, establishing the MPD as the expansion and phase II dose. Sixty-five additional patients received carfilzomib at the MPD. Adverse events included fatigue, nausea, anorexia, and dyspnea. Carfilzomib PK was dose proportional with a half-life <1 h. All doses resulted in at least 80 % proteasome inhibition in blood. Partial responses occurred in two patients in phase I, with 21.5 % stable disease after four cycles in evaluable patients in the expansion and phase II cohorts.

**Conclusion:**

Carfilzomib 20/36 mg/m^2^ was well tolerated when administered twice weekly by 2–10-min IV infusion. At this dose and infusion rate, carfilzomib inhibited the proteasome in blood but demonstrated limited antitumor activity in patients with advanced solid tumors.

## Introduction

The anticancer activity of proteasome inhibition has been validated clinically with bortezomib, a reversible dipeptide boronate proteasome inhibitor that primarily targets chymotrypsin-like (CT-L) activity but also inhibits caspase-like activity [1–3]. Studies have demonstrated the safety and efficacy of bortezomib in patients with hematologic malignancies, particularly multiple myeloma (MM) [4, 5], as well as mantle cell lymphoma [6], but have shown limited single-agent efficacy in solid tumors [7–10]. Preclinical data, including insufficient drug exposure beyond the exterior surface of certain solid tumors [11], physiologic stress responses that enhance solid tumor cell survival, and proteasome subunit composition that more closely resembles the immunoproteasome, have been cited to explain the limited clinical efficacy seen with bortezomib in solid tumors [12].

The selective proteasome inhibitor carfilzomib was recently approved in the USA for the treatment of relapsed and refractory MM [13]. Carfilzomib is an epoxyketone that is structurally and mechanistically distinct from bortezomib. It irreversibly inhibits the CT-L activities of both the constitutive proteasome and the immunoproteasome with minimal off-target activity [14, 15]. Preclinical and clinical studies have demonstrated that carfilzomib, unlike bortezomib [16], can be dosed on consecutive days, leading to prolonged and more complete inhibition of the proteasome [14, 17]. Stepped-up dosing and the infusion of carfilzomib intravenously over 2–10 min appeared to mitigate the infusion-like reaction seen with infusions of 1–2 min [17]. Mimicking the clinical dosing schedule, consecutive-day dosing of carfilzomib in a human solid tumor xenograft model demonstrated more effective antitumor activity than either bortezomib or carfilzomib dosed on days 1 and 4 [14]. In addition, in a study that replicated the clinical pharmacokinetics of both agents, a 1-h pulse of carfilzomib was more potent than bortezomib at killing solid tumor cell lines [14]. Taken together, these observations suggested that carfilzomib may have greater antitumor activity than bortezomib in solid tumors.

In the phase I and phase II study reported herein (NCT00531284), we assessed safety, tolerability, and efficacy of carfilzomib administered by 2–10-min IV infusion in patients with advanced solid tumors. The phase II tumor types were selected based on results from the phase I dose-escalation portion and preclinical data.

## Patients and methods

### Study objectives

The primary objective of the phase I dose-escalation portion of the study was to determine the maximum tolerated dose (MTD) of carfilzomib up to a maximum planned dose (MPD) of 20/36 mg/m^2^. The primary objective of the expansion (various tumor types) and phase II tumor-specific portions was to determine the disease control rate [DCR = complete response (CR) + partial response (PR) + stable disease (SD)] as defined by RECIST 1.0 [18] after four cycles of treatment at the MTD or MPD. Secondary objectives included pharmacokinetics (PK) and pharmacodynamics (PDn) of carfilzomib.

Patients 18 years or older with pathologically confirmed solid malignancies progressing after one or more prior regimens, and with evaluable or measurable disease according to the RECIST criteria, were eligible for the study. In addition, patients with NSCLC or ovarian cancer should have received one or more platinum-based chemotherapy regimens but three or fewer, or four or fewer total regimens, respectively, and SCLC patients three or fewer regimens. Renal cell cancer patients were required to have failed two or more prior regimens. Other eligibility criteria included life expectancy of at least 3 months, Eastern Cooperative Oncology Group performance status of 0–2, hemoglobin ≥8 g/dl, absolute neutrophil count ≥1,000/mm^3^, platelets ≥100,000/mm^3^, creatinine clearance (CrCl) >20 ml/min, bilirubin ≤1.5× upper limit of normal (ULN), and alanine transaminase (ALT) ≤3 × ULN. Patients with symptomatic brain metastases, comorbid severe medical conditions, New York Heart Association class III or IV congestive heart failure, acute active infection within 2 weeks of first dose, or neuropathy grade ≥2 by National Cancer Institute Common Terminology Criteria for Adverse Events (NCI CTCAE) v3.0 [19] at baseline were ineligible.

Institutional Review Board approval and written informed consent were obtained from each patient prior to any study-related procedure.

### Study drug administration

Carfilzomib (Onyx Pharmaceuticals, Inc, South San Francisco, CA) was administered by intravenous infusion over 2–10 min on days 1, 2, 8, 9, 15, and 16 of a 28-day cycle. For the phase I dose-escalation portion, the starting dose in all cohorts for days 1 and 2 of cycle 1 was 20 mg/m^2^, based on the tolerated dose identified in hematologic malignancies [19]. On all remaining cycle 1 dosing days (days 8, 9, 15, and 16) and in all subsequent cycles, carfilzomib was administered at a dose of 20 mg/m^2^ in cohort 1 (20/20 mg/m^2^), 27 mg/m^2^ in cohort 2 (20/27 mg/m^2^), and 36 mg/m^2^ in cohort 3 (20/36 mg/m^2^). Stepped-up dosing in cycle 1 and dexamethasone premedication were adopted to abrogate fever, chills and/or rigors, and dyspnea, all of which were infrequently observed during initial therapy in MM trials [17, 20–23]. Dexamethasone 4 mg (intravenous or oral) was administered prior to each carfilzomib dose for the first cycle and during subsequent cycles when indicated. Upon completion of 12 treatment cycles, patients had the option of enrolling in an open-label extension study, PX-171-010 (NCT00884312).

### Study design and determination of MTD

The phase I dose-escalation portion of the study followed the standard 3 + 3 design. Patients were enrolled at two sites in sequential cohorts of 3–6 patients each to establish the MTD. The MTD was defined as the highest dose up to the MPD at which at least one of six patients experienced treatment-related dose-limiting toxicities (DLT) during the first cycle. DLTs were defined as treatment-related ≥grade 2 neuropathy with pain, ≥grade 3 non-hematologic toxicity, grade 4 neutropenia or thrombocytopenia lasting 7 or more days, or thrombocytopenia with bleeding. Toxicity was graded according to NCI CTCAE v3.0 [19].

Once dose escalation was completed, an additional 11 evaluable patients with assorted tumor types could enroll in the expansion cohort to receive carfilzomib at the MTD/MPD. In the phase II tumor-specific portion of the study, cohorts of patients with NSCLC, SCLC, ovarian cancer, and renal cancer were to be treated at the MTD/MPD utilizing a Simon two-stage design. Tumor types included in the phase II tumor-specific cohorts were selected based on efficacy results from the phase I dose-escalation portion and preclinical data.

### Study assessments

#### Safety and efficacy

All patients who received at least one dose of study treatment were evaluable for the safety analysis. After the initiation of carfilzomib, adverse events (AEs) were evaluated at each visit. Patients completed a comprehensive neurologic evaluation and radiographic assessment of tumor burden using computerized tomography (CT) at baseline and every 8 weeks for the first 24 weeks, then every 12 weeks thereafter or at treatment discontinuation. Patients who received at least one cycle of treatment and had both baseline and post-baseline disease assessments were evaluable for efficacy. Treatment response was assessed according to RECIST 1.0 [18]. Patients with stable disease (SD) or an objective response (CR or PR) after two cycles continued treatment until disease progression or unacceptable toxicity.

#### Pharmacokinetics and pharmacodynamics

Pharmacokinetic properties of carfilzomib (maximum plasma concentration [C_max_], area under the plasma concentration versus time curve from time 0 to infinity [AUC_0–inf_], elimination half-life [t_1/2_], volume of distribution at steady-state [V_ss_], and plasma clearance [CL_p_]) were assessed for all patients in the phase I dose-escalation cohorts and for individuals at select sites in the expansion and phase II cohorts. Serial blood samples were collected on days 1 and 16 of cycle 1 and day 16 of cycle 2 as follows: predose, immediately post-dose, and then at 5, 15, and 30 min, and 1, 2, and 4 h post-dose. Plasma samples for PK studies were assayed using validated liquid chromatography/mass spectrometry with a lower limit of detection of 0.10 ng/ml [17].

Pharmacodynamics was assessed for all patients in the phase I dose-escalation portion and patients at select sites in the expansion and phase II portions of the study. Whole blood was collected on day 1 of the first two cycles prior to and 1 h after infusion for measurement of proteasome activity in whole blood and peripheral blood mononuclear cells (PBMCs). CT-L activity was assessed using a fluorogenic substrate assay with succinyl–Leu–Leu–Val–Tyr–AMC (LLVY) as previously described [15, 19].

### Statistical analysis

Continuous and categorical data were summarized with descriptive statistics or frequencies and percentages, respectively. For the phase II tumor-specific portion, the Simon two-stage sample size calculation applied a null hypothesis objective response rate (ORR = CR + PR) of 2 % and an alternative hypothesis ORR of 15 %, with the type I error at 5 % and a power of 0.8. For stage 1, 11 evaluable patients were to be enrolled in each of the four solid tumor cohorts. If one or more patients demonstrated an objective response (CR or PR), that cohort would proceed to the second stage and enroll a total of 37 evaluable patients. Because there were multiple study sites, over enrollment was permitted.

Pharmacokinetic parameters were estimated by non-compartmental analysis using WinNonlin Enterprise Version 5.2 (Pharsight Inc., Mountain View, CA). Dose proportionality was assessed using a one-way analysis of variance (ANOVA) at α = 0.05 significance level, with Bonferroni’s multiple comparison for post-tests (SAS Institute, Inc., Cary, NC).

## Results

### Patients

Fourteen patients were enrolled in the phase I dose-escalation portion, and 65 patients were enrolled in the expansion (*n* = 16) and phase II tumor-specific cohorts (*n* = 49) between September 2007 and December 2009. Patient demographics and baseline characteristics are presented in Table 1. Most patients were heavily pretreated, with a median of three (range 1–9) prior chemotherapy regimens.

**Table 1:** Baseline characteristics and treatment history

Characteristics and treatment history	Phase I dose escalation (*n* = 14)	Expansion and phase II^c^ (*n* = 65)
Gender, *n* (%)		
Female	6 (43)	38 (59)
Male	8 (57)	27 (42)
Age, years, median (range)	59.5 (36–75)	62 (41–87)
Tumor type, *n*		
Small cell lung	3	9
Non-small cell lung	2	15
Renal	1	10
Ovarian	1	15
Other^a, b^	7	16
Number of prior therapies, median (range)	3 (2–6)	3 (1–9)
Prior platinum-based regimens, *n* (%) Prior taxanes, *n* (%)	12 (85) 7 (50)	49 (75) 35 (54)

^a^Phase I dose-escalation other tumors included: (one patient each) colon, gastric, mesothelioma, oropharynx, pharyngeal, soft palate and tonsil, and sarcoma

^b^Expansion other tumors included: (two patients each) colon, endometrial, esophageal, pancreas, and thyroid; and (one patient each) anal, cervical, gall bladder carcinoma, prostate, squamous cell carcinoma, and vulvar

^c^Enrollment to phase II tumor-specific cohorts required 11 evaluable patients for stage 1 of the Simon two-stage design. None of the cohorts proceeded to stage 2: NSCLC and ovarian cohorts did not meet stage 2 criteria, and SCLC and renal cohorts were terminated before stage 1 enrollment was completed

In the phase I dose-escalation portion, a median of 2 cycles (range 1–12) were administered. No patients had their carfilzomib dose reduced due to AEs. During dose escalation, one patient completed 12 cycles of therapy and rolled over into the open-label extension study PX-171-010, nine of 14 patients (64.3 %) discontinued the study due to disease progression, two patients (14.3 %) discontinued due to AEs, one patient (7.1 %) discontinued due to lack of clinical benefit, and one patient (7.1 %) withdrew consent.

In the expansion and phase II portions, a median of two cycles (range 1–12) were administered. Twenty-one patients (32.3 %) missed at least one carfilzomib dose due to AEs (29.2 %) or schedule conflict (3.1 %), and the carfilzomib dose was reduced for eight patients (12.3 %) due to AEs of elevated blood creatinine/decreased CrCl. Two patients completed 12 cycles of carfilzomib therapy and continued treatment in the extension study. Forty-two patients (64.6 %) discontinued treatment due to disease progression, seven (10.8 %) discontinued due to an AE, four (6.2 %) withdrew consent, and 10 (15.4 %) cited “other” reasons for discontinuing.

### DLT and MTD

No MTD was identified up to the MPD in the phase I dose-escalation portion. There were no DLTs in cohorts 1 (*n* = 3) and 2 (*n* = 4). In cohort 3 (*n* = 7), a patient with gastric cancer experienced a single DLT (grade 3 fatigue) during cycle 1 at 20/36 mg/m^2^. Because no additional patients experienced DLTs at this dose level, 20/36 mg/m^2^, the MPD was recommended for the expansion and phase II tumor-specific portions.

### Safety

All 79 patients received at least one dose of carfilzomib and were evaluable for safety, and all experienced at least one AE of any grade (Table 2).

**Table 2:** Most common adverse events and treatment-related adverse events

	Phase I dose escalation			Expansion and phase II
	Cohort 1 (20/20 mg/m^2^) (*n* = 3)	Cohort 2 (20/27 mg/m^2^) (*n* = 4)	Cohort 3 (20/36 mg/m^2^) (*n* = 7)	Cohorts (20/36 mg/m^2^) (*n* = 65)
Any adverse event, *n* (%)	3 (100)	4 (100)	7 (100)	65 (100)
Fatigue	0	2 (50.0)	3 (42.9)	38 (58.5)
Nausea	0	0	4 (57.1)	28 (43.1)
Anorexia	0	0	2 (28.6)	26 (40.0)
Dyspnea	0	0	1 (14.3)	24 (36.9)
Diarrhea	1 (33.3)	0	3 (42.9)	18 (27.7)
Vomiting	0	0	4 (57.1)	18 (27.7)
Pyrexia	0	0	3 (42.9)	18 (27.7)
Chills	0	0	2 (28.6)	18 (27.7)
Anemia	0	1 (25.0)	1 (14.3)	17 (26.2)
Any Grade 3/4 adverse event, *n* (%)	1 (33.3)	2 (50.0)	5 (71.4)	44 (67.7)
Lymphopenia	0	1 (25.0)	3 (42.9)	10 (15.4)
Anemia	0	1 (25.0)	0	4 (6.2)
Fatigue	0	0	1 (14.3)	4 (6.2)
Any treatment-related adverse event, *n* (%)	2 (66.7)	2 (50.0)	6 (85.7)	58 (89.2)
Fatigue	0	2 (50.0)	3 (42.9)	24 (36.9)
Nausea	0	0	4 (57.1)	21 (32.3)
Vomiting	0	0	4 (57.1)	12 (18.5)
Anemia	0	0	1 (14.3)	14 (21.5)
Chills	0	0	1 (14.3)	14 (21.5)
Any grade 3/4 treatment-related adverse event, *n* (%)	0	0	2 (28.6)	20 (30.8)
Lymphopenia	0	0	1 (14.3)	6 (9.2)
Anemia	0	0	0	4 (6.2)
Hypophosphatemia	0	0	0	3 (4.6)
Renal failure acute	0	0	0	2 (3.1)

In the phase I dose-escalation portion, the most common AEs were headache (42.9 %), fatigue (35.7 %), and hypokalemia (35.7 %). The AEs leading to discontinuation for two patients in the dose-escalation cohorts—aspiration pneumonia (one patient in cohort 2) and diarrhea (one patient in cohort 3)—were not among the most common treatment-related AEs overall.

The most frequently reported AEs of any grade in the expansion and phase II tumor-specific portions were fatigue (58.5 %), nausea (43.1 %), anorexia (40.0 %), and dyspnea (36.9 %) (Table 2). Grade ≥2 peripheral neuropathy and any grade hepatotoxicity were not observed. Ten patients (15.4 %) experienced grade 3 lymphopenia without clinical sequelae; other grade 3/4 AEs were infrequent. The most common carfilzomib-related AEs in the expansion and phase II portions included fatigue (36.9 %), nausea (32.3 %), anemia (21.5 %), and chills (21.5 %). Seven patients discontinued primarily due to the following AEs (one patient each): congestive heart failure, hyponatremia, infusion-related reaction, pneumonia/septic shock, spinal cord compression, malignant pleural effusion, and neuropathy. There were four deaths on study or within 30 days of ending treatment, all due to PD. No deaths were considered related to carfilzomib.

### Pharmacokinetics and pharmacodynamics

**Fig. 1:**
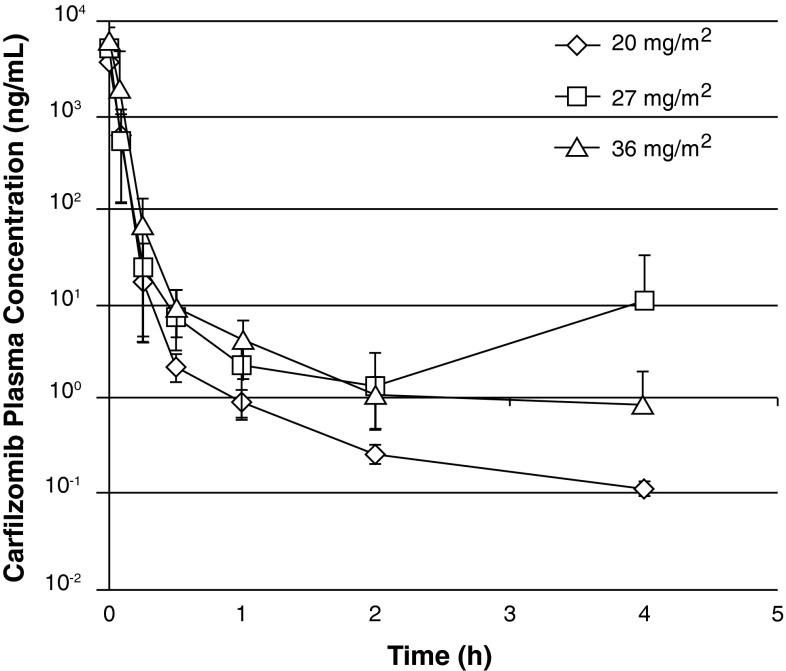
Plasma concentration versus time profiles for carfilzomib. Plasma concentration of carfilzomib at various time points after a 2–10-min IV infusion of 20, 27, or 36 mg/m^2^ on day 16 of cycle 1

Samples were collected from all 14 phase I dose-escalation patients and from 16 patients in the expansion and phase II tumor-specific cohorts. Patients with incomplete samples were excluded from PK calculations and dose-proportionality assessments. At all doses, carfilzomib plasma concentrations declined rapidly following 2–10-min IV infusion; by 4 h after dosing, plasma levels were below the limit of detection in almost all patients (Fig. 1). C_max_ and AUC increased proportionally across the three doses tested, and the half-life was approximately 1 h or less in all cohorts (Table 3).

**Table 3:** Pharmacokinetic parameters of carfilzomib in cycle 1

Days	Dose (mg/m^2^)	*N*	C_max_ (ng/mL)^a^	*t*_1/2_ (h)^b^	CL (L/h)^c^	*V*_ss_ (L)^c^	AUC_last_ (ng h/mL)^a^
1	20	30^d^	2390 ± 104	0.44 (0.15–2.20)	263 ± 398	27.7 ± 48.6	251 ± 92.0
16	20	3	3410 ± 51.5	1.10 (1.00–1.13)	136 ± 52.8	7.75 ± 3.77	269 ± 61.7
16	27	5^e, f^	4232 ± 48.8	0.35 (0.26–0.92)	150 ± 30.9	11.1 ± 4.45	379 ± 24.8
16	36	13^g^	5718 ± 46.5	0.87 (0.38–1.81)	116 ± 48.6	9.33 ± 4.80	594 ± 52.5

^a^Geometric mean ± coefficient of variation  %

^b^Median (range)

^c^Arithmetic mean ± SD

^d^ *t*_1/2_, *V*_ss_, *CL* *n* = 23

^e^AUC_inf_, *t*_1/2_, *V*_ss_, *CL* *n* = 4

^f^Includes 1 patient from the phase 2 20/36 cohort whose dose was reduced

^g^ *t*_1/2_, *V*_ss_, *CL* *n* = 10

**Fig. 2:**
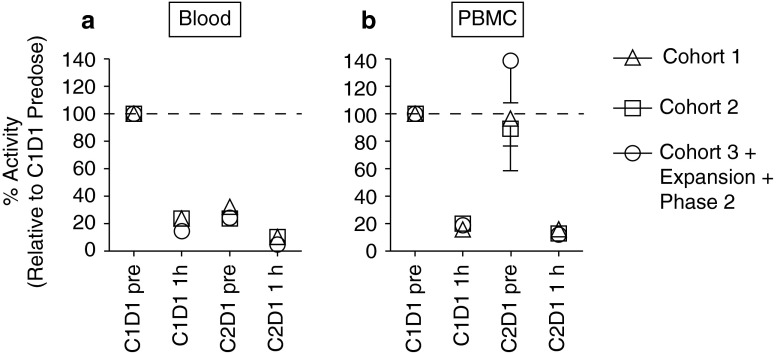
Proteasome activity in whole blood and peripheral blood mononuclear cells. Chymotrypsin-like-specific proteasome activity in whole blood (**a**) and PBMCs (**b**) prior to and 1 h after the first dose in cycle 2 using LLVY-AMC substrate. Data are presented as mean activity (±SEM) relative to cycle 1 day 1 predose samples. (**c**) cycle; (**d**) day; pre, pre-dose

One hour after administration of carfilzomib on day 1 of cycle 2, proteasome CT-L activity in whole blood and PBMCs was inhibited by ≥80 % (Fig. 2). Median proteasome inhibition was 83.4 % (range 82.9–85.5 %), 91.6 % (range 79.0–93.2 %), and 88.6 % (range 54.7–99.4 %) with carfilzomib 20, 27, and 36 mg/m^2^, respectively. Minimal recovery of constitutive proteasome activity was observed between cycles in whole blood, but there was recovery in PBMCs by the start of the second cycle.

### Antitumor activity

Twelve of 14 patients (85.7 %) in the phase I dose-escalation portion and 51 of 65 patients (78.5 %) in the expansion and phase II tumor-specific cohorts were evaluable for response. Two patients in the dose escalation and 14 patients in the expansion and phase II tumor-specific cohorts (17.7 % overall) either did not have a post-baseline disease assessment, received less than one cycle of therapy, or both.

In the phase I dose-escalation portion, PR was confirmed in two patients treated with carfilzomib at 20/36 mg/m^2^. A patient with renal cancer who had failed two multi-targeted antiangiogenesis inhibitors and an mTOR inhibitor had a >80 % reduction in tumor burden after five cycles of carfilzomib and continued on therapy for 13 cycles until disease progression with a dural-based brain metastasis. A patient with SCLC who had received six prior chemotherapy regimens had a durable PR with carfilzomib and remained on treatment for 39 cycles until disease progression. Two additional patients achieved SD, and eight patients had PD.

**Fig. 3:**
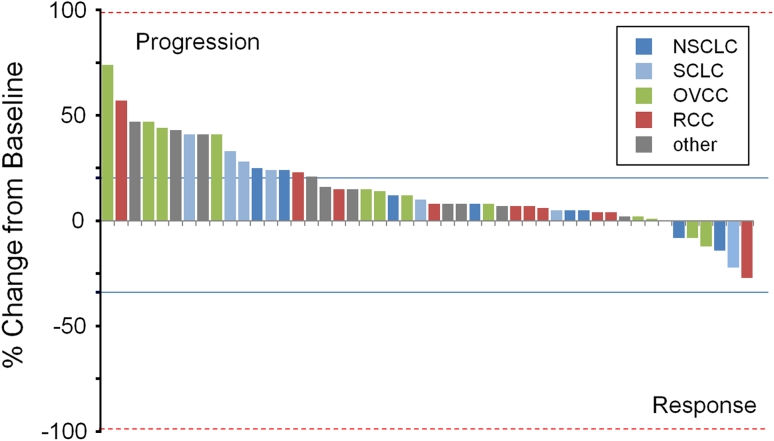
Maximum reduction in target lesions in expansion and phase II patients. Percentage change in target lesion from baseline as assessed by CT scan. According to RECIST criteria, best overall responses are assigned after confirmation in a subsequent scan after ≥4 weeks; therefore, maximum decrease in target lesion may differ from best overall response. Presented are the 48 patients treated at the MTD in the expansion and phase II portions, with measureable disease evaluable by RECIST. *Solid lines* represent thresholds for response: 20 % increase for progressive disease and 30 % decrease for partial response. NSCLC, non-small cell lung cancer; SCLC, small cell lung cancer; OVCC, ovarian cancer; RCC, renal cell cancer

Among the 51 evaluable patients in the expansion and phase II tumor-specific cohorts, 48 (94.1 %) had measurable disease according to RECIST criteria. No patients achieved PR or better after four cycles of therapy or over the study period. However, 22 patients (43.1 %) had SD after two cycles and 11 of these patients had SD over four cycles (primary endpoint, overall DCR = 21.5 %). Of four patients with SD for 6 months or longer, two patients with renal clear cell carcinoma had SD for 6 months, a patient with NSCLC had SD for 10 months, and a patient with ovarian cancer had 12 months of SD. PD was the best response in all other expansion and phase II patients (29/51, 56.9 %). None of the phase II tumor-specific cohorts proceeded to stage 2 enrollment. Maximum percent change in radiographic assessment of target lesions for dose expansion and phase II patients is shown in Fig. 3.

## Discussion

In the current study, carfilzomib was safely administered by 2–10-min intravenous infusion at the MPD of 20/36 mg/m^2^ in patients with advanced solid malignancies. This dose exceeds that of 20/27 mg/m^2^ determined as the tolerable dose for 2–10-min intravenous infusion in patients with MM [17, 20–23]. Although all patients experienced AEs, carfilzomib was generally well tolerated with few patients reducing the dose or discontinuing therapy due to AEs. Mild or moderate non-hematologic AEs including fatigue, nausea, and anorexia were most common and similar to AEs observed in patients with MM [17, 20–23]. Notable in these pretreated patients, many with prior taxane and platinum-based therapy exposure, was the absence of treatment-emergent grade ≥2 peripheral neuropathy, consistent with earlier observations [20–23]. In contrast to results in patients with MM, cardiopulmonary, hepatic, and renal toxicities were not common in patients with solid tumors.

The primary endpoint of the expansion and phase II tumor-specific portions of the study was DCR after four cycles. Although no patients achieved PR or better during this part of the study, 11 of 51 evaluable patients (21.5 %) with advanced solid tumors experienced SD after four cycles of carfilzomib, with four of these patients achieving SD for 6 months or more.

Despite demonstrating significant proteasome inhibition in surrogate tissue at the highest dose tested (36 mg/m^2^) and PK results consistent with previous observations, the efficacy results seen in patients with advanced solid tumors differ from the robust antitumor activity seen with lower doses of carfilzomib in MM [20–23]. As noted, the proteasome inhibitor bortezomib has shown disappointing single-agent activity and limited activity in combination regimens in a variety of solid tumors [7–10, 24]. Despite a large volume of distribution, it is possible that carfilzomib does not effectively penetrate solid tumors when infused over 2–10 min, especially given its rapid systemic clearance and very short elimination half-life. Proteasome inhibition in PBMCs was rapid and prolonged; however, the PBMCs likely do not represent the amount of inhibition within the tumor cells. It is also possible that, despite irreversible binding of carfilzomib to the proteasome, recovery of proteasome activity between doses was sufficient to permit survival of solid tumor cells. Antitumor activity in a small number of patients suggests that effective levels were achieved in some tumors or that some tumors were more sensitive to the effects of proteasome inhibition. Future studies with paired pre- and post-treatment tumor biopsies may provide a more accurate determination of the amount and duration of proteasome inhibition within the tumor, reveal mechanisms of resistance, and assist in identifying biomarkers predictive of response to carfilzomib in solid tumors.

The stipulation of a protocol-defined MPD restricted further dose escalation, which may have contributed to the low response rate. The design of the current study and prespecified MPD was based on the safety and efficacy data generated from a phase 1 trial of carfilzomib in patients with hematologic malignancies [17]. In that study, carfilzomib was well tolerated, but doses >20/27 mg/m^2^ were not explored. As there was concern regarding tolerability of higher doses and since the PD endpoint of >90 % proteasome inhibition was achieved in PMBCs at 20/27 mg/m^2^ [17], the decision was thus made to define a MPD of 20/36 mg/m^2^ for this study. While the study was ongoing, emerging preclinical data suggested that a longer infusion time might result in better tolerance and allow administration of higher doses of carfilzomib over longer periods, with potential for greater and more prolonged proteasome inhibition and improved efficacy [25]. With the phase II NSCLC and ovarian cohorts not meeting criteria to proceed to the second stage, and the low likelihood that criteria would be met in the remaining two tumor-specific cohorts, patient accrual was terminated to pursue a currently ongoing exploration of a 30-min intravenous infusion.

In summary, the MPD of carfilzomib, 20/36 mg/m^2^, was well tolerated when administered by 2–10-min IV infusion on a twice weekly schedule. At this dose and infusion rate, carfilzomib demonstrated a PK profile consistent with previous reports in patients with MM and inhibited the proteasome in blood, but demonstrated limited antitumor activity in patients with advanced solid tumors. The tolerable AE profile—in particular, the lack of clinically meaningful peripheral neuropathy with carfilzomib—provides the opportunity for combination studies with other cytotoxic or targeted agents that might leverage the additive or synergistic effects of proteasome inhibition. Whether higher doses and/or combinations with cytotoxic or targeted agents could lead to improved efficacy with an acceptable safety profile remains an open question worthy of further investigation.
